# Association between nutritional inflammation index and diabetic foot ulcers: a population-based study

**DOI:** 10.3389/fnut.2025.1532131

**Published:** 2025-01-24

**Authors:** Zhou Lin, Wanli Zhuang, Lei Wang, Weifeng Lan

**Affiliations:** ^1^Department of Burns and Plastic Surgery, Longyan First Affiliated Hospital of Fujian Medical University, Longyan, Fujian, China; ^2^Department of Gastroenterology, Jinjiang Municipal Hospital, Shanghai Sixth People’s Hospital Fujian Campus, Quanzhou, Fujian, China; ^3^Department of Science and Education, Longyan First Affiliated Hospital of Fujian Medical University, Longyan, Fujian, China

**Keywords:** albumin/neutrophil to lymphocyte ratio, diabetic foot ulcers, NHANES, inflammation, nutrition

## Abstract

**Background:**

Diabetic foot ulcers (DFU), a frequent complication of the worldwide disease (diabetes), are the primary causes of amputations and early mortality. The development of DFU is inseparably linked with inflammation and nutrition, necessitating a comprehensive evaluation of their impact on DFU risk. This study aimed to establish a new predictive metric that integrated immune inflammation and nutritional markers to holistically assess the risk of DFU development.

**Methods:**

Data were sourced from NHANES, extracting participant from 1999 to 2004. Analysis of multivariate logistic regression and restricted cubic spline were employed to elucidate the connection and non-linear relationship between albumin/neutrophil to lymphocyte ratio (ANLR) and DFU. Stratified subgroup analysis identified advantageous populations, while interaction analysis evaluated variable interactions with ANLR. These approaches collectively contributed to a sensitivity analysis, improving the reliability of the outcomes.

**Results:**

Out of 29,608 participants extracted, 1,531 qualified based on the study criteria. Employing the ANLR low group as a reference, the high group demonstrated a 54% reduction in DFU risk. Every increase of 0.1 unit in ANLR correlated with a 5% decrease in DFU risk. Moreover, an L-shaped non-linear link was observed. The turning point was at 3.09. Left of the inflection point, the relationship was negatively correlated. Beyond this point, further increased in ANLR no longer decrease DFU risk.

**Conclusion:**

The study not only proposed a new comprehensive indicator for predicting DFU for the first time but also specified the impact of ANLR on DFU risk. Broadly, a negative correlation existed between the two. Yet, a detailed analysis revealed that this negative correlation involved an inflection point effect. Furthermore, the study investigated how dynamic changes in ANLR affect DFU risk, aiding clinicians in more accurately assessing individual DFU risk and facilitating earlier identification and intervention of DFU. Therefore, for diabetic patients with low serum albumin, appropriate supplementation of albumin was crucial. Additionally, maintaining the NLR at an appropriate level should not be overlooked. Given the components of ANLR were widely used and readily available in clinical settings, their future clinical applications hold great potential.

## Introduction

As a globally acknowledged health problem, diabetes presents considerable challenges to public health and significantly affects individuals’ quality of life. Allowing diabetes to progress without intervention often leads to a range of complications such as diabetic foot ulcers (DFU), diabetic nephropathy, and diabetic retinopathy (1). DFU affected millions around the world, having a global incidence rate of roughly 6.3% (2, 3). Notably, DFU and amputation were major reasons for significant declines in life quality and even early death (4). Therefore, early identification of DFU was of great importance.

Studies demonstrated that DFU was closely associated with chronic inflammation. Higher incidence rates of DFU occurred in populations with elevated inflammation levels, and continuous inflammation could aggravate ulcers, resulting in non-healing wounds and, in extreme cases, amputation (5–9). Previous research has revealed that the onset and progression of DFU correlate with albumin levels (a common and widely applied indicator for assessing nutritional status). The lower the albumin, the higher the probability of DFU and the slower the healing of wounds (10–12). Furthermore, inflammation and albumin were not isolated factors. They could mutually influence each other. Inflammation could affect albumin levels through TNF-*α* and CRP, while albumin has been proven to have anti-inflammatory effects (13–15). Therefore, assessing DFU occurrence solely based on inflammation or albumin levels was insufficient. A new, comprehensive indicator was urgently needed to assess both inflammation and nutritional status effectively.

Albumin/neutrophil to lymphocyte ratio (ANLR), consisting of neutrophils to lymphocytes ratio (NLR) and albumin, represented a new integrated index for evaluating inflammation and nutrition in DFU, specifically formulated as the albumin/NLR. Past research has demonstrated a close correlation between NLR and inflammation, suggesting its use as a predictive indicator of inflammation. An elevated NLR signified a greater level of inflammation. NLR was widely used in the fields of oncology, diabetes, and cardiovascular diseases (16–19). Historical data showed that NLR was an independent risk factor for the onset and development of DFU, and it assessed inflammation levels in DFU patients effectively (20, 21). Albumin, commonly used to evaluate nutritional status, has been shown to have a strong connection with the incidence, recurrence, and amputation risk of DFU (10, 12, 22). Drawing on the evidence provided, this research constructed the ANLR to thoroughly and systematically evaluate how inflammation and nutritional status influence the risk of DFU onset.

This study has two main goals. One goal was to employ the novel indicator, ANLR, to evaluate the risk of DFU and explore any non-linear relationships between them. The other was to study the impact of dynamic changes in ANLR on DFU risk, assisting clinicians in more accurately predicting DFU risk and facilitating personalized treatment and early intervention.

## Methods

### Study participants

The study’s data were derived from the National Health and Nutrition Examination Survey (NHANES), a comprehensive, multi-center, multi-stage sampling database that provided representative data on American health and nutrition, accessible freely and publicly. For more details, visited website^1^. From 1999 to 2004 cohorts, data on 29,608 participants were extracted. Initially, 19,652 participants with indeterminate DFU status were excluded. Additionally, the study targeted diabetic individuals aged 20 and above, resulting in the exclusion of 8,066 participants. Lastly, 359 participants lacking information on essential variables [including albumin, neutrophils, lymphocytes, gender, race, smoking or alcohol status, hypertension, cardiovascular disease (CVD), and HbA1c] were excluded. In the end, 1,531 participants qualified for the study.

### Variable determination

**Figure 1:**
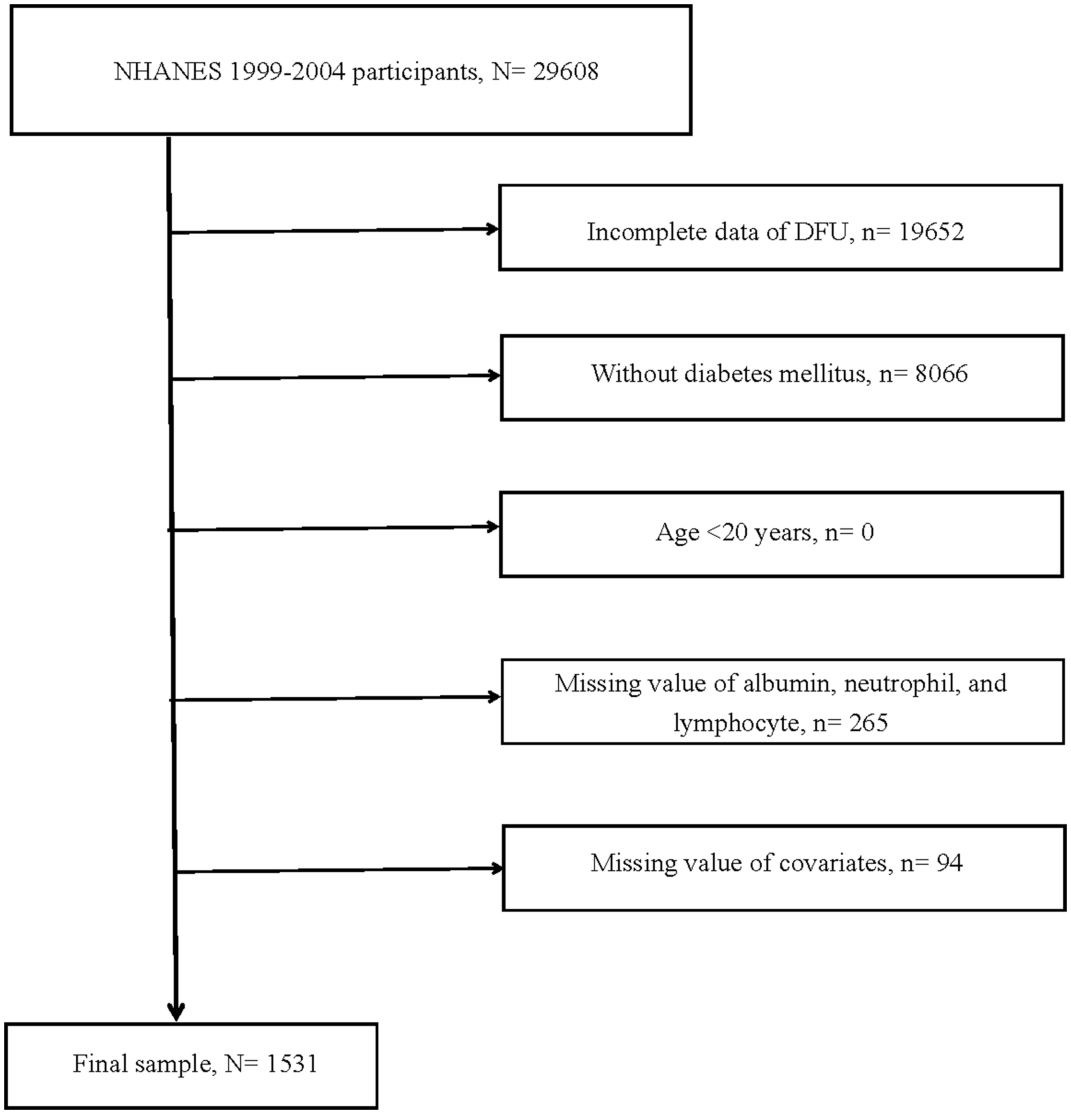
Flow chart of study participants. DFU, diabetic foot ulcers; N, the number of patients being included; n, the number of patients being excluded.

**Figure 2:**
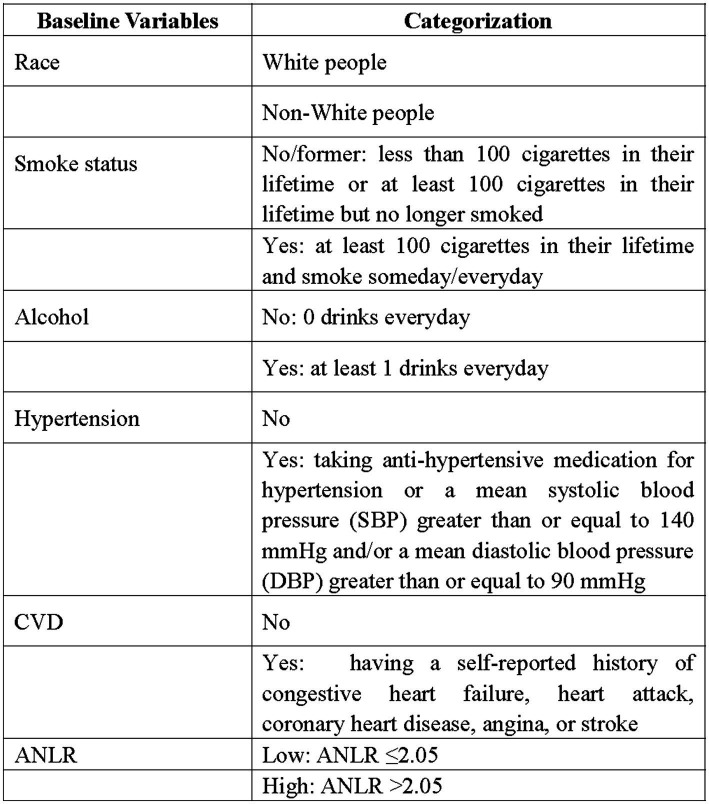
Grouping and categorization of baseline variables. CVD, cardiovascular disease.

The NLR was derived from the ratio of neutrophils to lymphocytes. ANLR was composed of albumin/NLR. Diabetes was characterized as follows: (1) Fasting blood glucose ≥7.0 mmol/L. (2) Random blood glucose ≥11.1 mmol/L. (3) HbA1c ≥6.5%. (4) Administration of antidiabetic drugs. (5) Having been diagnosed with diabetes by a physician. Fulfilling any of these criteria was considered as having diabetes. DFU was identified through survey findings. Specifically, diabetic patients with unhealed ulcers on lower limbs for over 4 weeks were categorized as DFU. Moreover, baseline features including race, gender, age, alcohol, smoking, CVD (including coronary heart disease, congestive heart failure, heart attack, stroke, and angina.), hypertension, and HbA1c were retrieved from NHANES. Figure 1 provided a detailed depiction of the study’s procedure. Figure 2 offered a specific categorization of the variables.

### Statistical methods

Following NHANES guidelines, this study took into account sample weights, clustering, and stratification, conducting statistical analyses with R4.3.1. The variance in continuous baseline variables was clarified through the *t*-test, with the distinctions in categorical variables were discerned by the chi-squared test. The relationship between ANLR and the risk of DFU was established using both univariate and multivariate logit analyses. In order to more accurately demonstrate the effects of various covariates on DFU risk, this research formulated three models, analyzing from multiple perspectives. The crude model incorporated only ANLR, disregarding the impact of other covariates. Model1 involved demographic variables (gender, age, race) as modifiers in the multivariate logit analysis to specify the effect of ANLR on DFU risk after demographic adjustments. Model2 integrated demographic variables, lifestyle habits (smoking and drinking), previous medical history (hypertension and CVD), and clinical laboratory indices (HbA1c), providing a more accurate analysis of the impact of ANLR on DFU risk across these four dimensions. This model was also of primary interest in this research. Notably, ANLR was further considered a continuous variable instead of a categorical one in the multivariate logit analysis. This approach examined the impact of each 0.1 unit change in ANLR on DFU risk, thereby enabling doctors to more effectively quantify the risk of DFU. Moreover, in light of a possible non-linear relationship between ANLR and DFU risk, this study utilized restricted cubic spline (RCS) analysis to ascertain their non-linear interactions and pinpoint inflection points. Simultaneously, segmented logit analysis was used to clarify threshold effects. Stratified and interaction analyses served as the sensitivity analyses in this research, designed to boost the reliability of discoveries. Continuous factors like age and HbA1c were classified as categorical variables in stratified analysis, helping to identify advantageous subpopulations. Through interaction analysis, the impact of different covariates on ANLR was evaluated.

### Ethics statement

National Center for Health Statistics Ethics Review Committee sanctioned human studies from NHANES. The informed consent forms were provided by participants. Detailed information was listed in website^2^.

## Results

### Baseline features

There were 1,531 participants who met the inclusion criteria. Among them, 121 individuals (7.98%) had DFU. For these 1,531 participants, the average age was 61.63 years and the average ANLR was 2.21. The low and high groups displayed different baseline characteristics, as observed in Table 1. Put differently, the high group included younger individuals, fewer White people, a lower proportion of males, and lower NLR and neutrophil counts. Meanwhile, the high group has higher albumin and lymphocyte counts and a greater proportion with a history of CVD. Other aspects, such as smoking and drinking status, hypertension history, and HbA1c, showed no significant differences between the groups. More exhaustive details were presented in Table 1.

**Table 1:** Baseline characteristics of the study population.

Characteristics	ANLR			
	Total	Low 1.47 [0.22,2.05]	High 2.71 [2.05,11.50]	*P*
Participants, *n*	1,531	765	766	
ANLR, mean	2.21 (2.15,2.28)	1.43 (1.40,1.46)	3.00 (2.91,3.09)	<0.0001
Age, year	61.63 (60.79,62.47)	62.89 (61.80,63.98)	60.37 (59.30,61.44)	<0.001
Gender, *n* (%)				0.04
Male	819(52.57)	448(56.38)	371(48.75)	
Female	712(47.43)	317(43.62)	395(51.25)	
Race, *n* (%)				<0.0001
White people	630(67.13)	364(72.82)	266(61.43)	
Non-white people	901(32.87)	401(27.18)	500(38.57)	
Albumin, g/dL	4.19 (4.17,4.22)	4.13 (4.09,4.16)	4.26 (4.23,4.29)	<0.0001
NLR, mean	2.37 (2.27,2.47)	3.21 (3.07,3.36)	1.52 (1.49,1.56)	<0.0001
Neutrophil, K/μL	4.52 (4.41,4.63)	5.19 (5.05,5.33)	3.84 (3.72,3.96)	<0.0001
Lymphocyte, K/μL	2.18 (2.12,2.24)	1.76 (1.70,1.81)	2.60 (2.53,2.68)	<0.0001
HbA1c, %	7.24 (7.13,7.36)	7.15 (6.97,7.32)	7.34 (7.19,7.49)	0.11
Smoke status, *n* (%)				0.46
No/former	1,282(82.00)	634(81.01)	648(83.00)	
Yes	249(18.00)	131(18.99)	118(17.00)	
Alcohol, *n* (%)				0.3
No	862(53.58)	428(52.03)	434(55.14)	
Yes	669(46.42)	337(47.97)	332(44.86)	
Hypertension, *n* (%)				0.09
No	396(29.13)	179(26.31)	217(31.95)	
Yes	1,135(70.87)	586(73.69)	549(68.05)	
CVD, *n* (%)				0.01
No	1,099(72.89)	505(69.28)	594(76.51)	
Yes	432(27.11)	260(30.72)	172(23.49)	
DFU, *n* (%)				0.004
No	1,410(92.02)	691(89.26)	719(94.79)	
Yes	121(7.98)	74(10.74)	47(5.21)	

### Logit analysis

Table 2 listed the results of the association between ANLR and DFU. Univariate analysis from the crude model demonstrated that a low ANLR was a risk factor for DFU. Model2 included factors such as gender, age, race, smoking and drinking status, and histories of CVD and hypertension, along with HbA1c as adjustments, and it indicated a negative correlation between DFU risk and ANLR—that was, a higher ANLR corresponded to a lower DFU risk (a 54% reduction in risk). Furthermore, each 0.1 unit increase in ANLR resulted in a 5% reduction in DFU risk, at 0.95 (0.92–0.99).

**Table 2:** Relationships between ANLR and DFU.

ANLR	OR, 95%CI		
	Crude	Model 1	Model 2
Low	ref	ref	ref
High	0.46(0.27,0.77)	0.46(0.26,0.79)	0.46(0.27,0.81)
Per 0.1U increment	0.95(0.92,0.99)	0.95(0.92,0.99)	0.95(0.92,0.99)
*P* for trend	0.005	0.010	0.010

ANLR, albumin/neutrophil to lymphocyte ratio; DFU, diabetic foot ulcers; CVD, cardiovascular disease.

Model 1: adjusted for ANLR+ age, gender, and race. Model 2: model 1+ adjusted for smoke status, alcohol, hypertension, CVD, and HbA1c (%).

### Non-linear association

**Figure 3:**
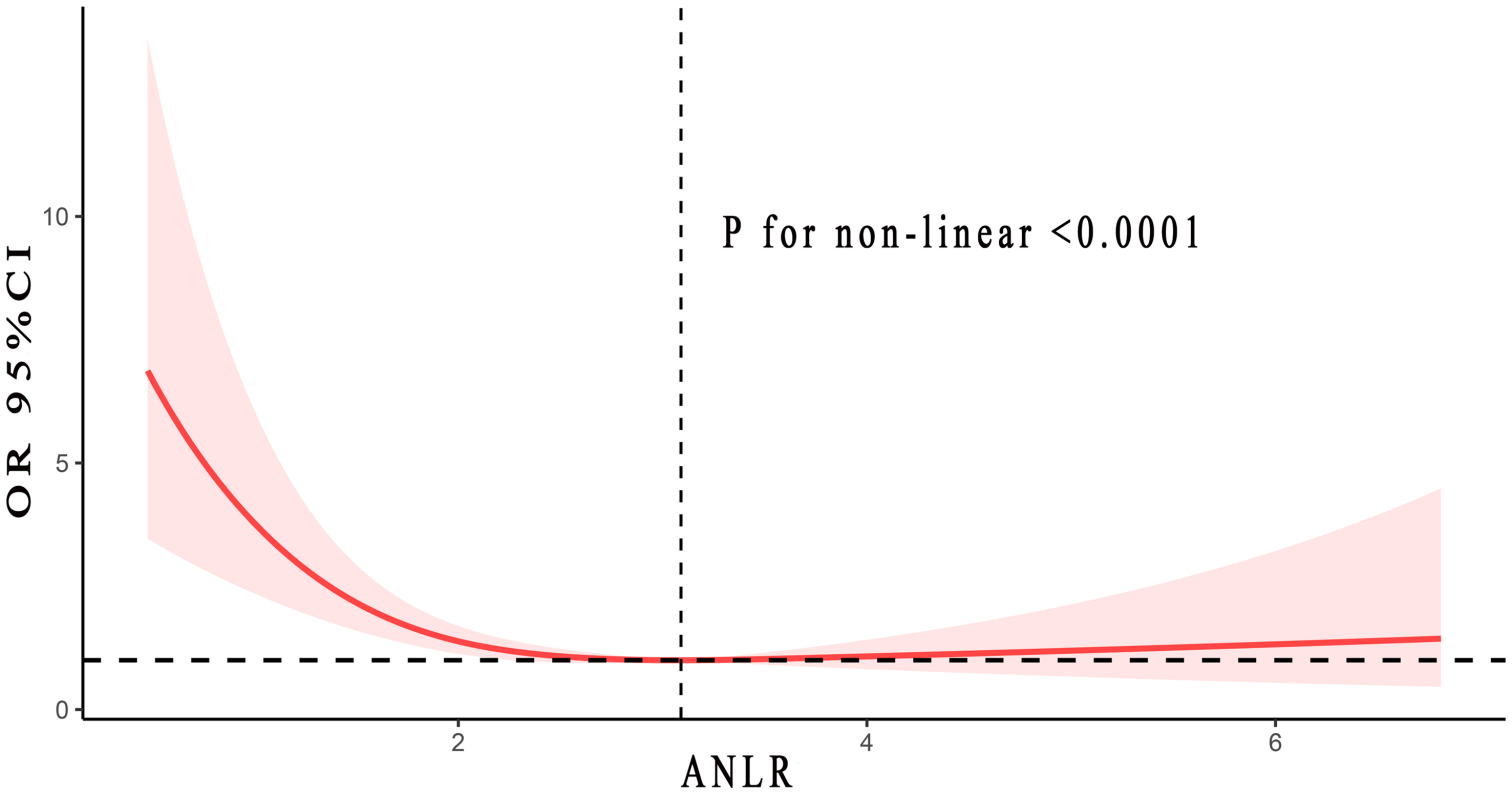
Non-linear relationship between ANLR and DFU. Adjusted for age, gender, race, smoke and alcohol status, hypertension, CVD, and HbA1c. The solid and red shadow represented the estimated values and their 95% CIs, respectively. ANLR, albumin/neutrophil to lymphocyte ratio; DFU, diabetic foot ulcers; CVD, cardiovascular disease.

Figure 3 listed the non-linear association between ANLR and DFU risk, revealing an L-shaped pattern (*p* < 0.001). In other words, the risk of DFU did not uniformly decrease with increasing ANLR, rather, there was an inflection point at 3.09. Specifically, when ANLR was below 3.09, the risk of DFU decreased as ANLR increased, with each 0.1 unit increase reducing the risk by 8% (*p* = 0.002) On the right side of the inflection point, when ANLR exceeded 3.09, the risk of DFU did not vary with further increased in ANLR but remained stable (*p* = 0.44). In summary, as ANLR increased, the risk of DFU correspondingly decreased. However, this marked downward trend stabilized after the inflection point (3.09). Detailed information was showed in Table 3.

**Table 3:** Threshold effect analysis of ANLR on DFU.

	Incidence	
	Per 0.1 U increment	*P*
<3.09	0.92(0.87,0.97)	0.002
>3.09	0.99(0.96, 1.02)	0.44

ANLR, albumin/neutrophil to lymphocyte ratio; DFU, diabetic foot ulcers; CVD, cardiovascular disease.

Model was adjusted for ANLR, age, gender, race, smoke status, alcohol, hypertension, CVD, and HbA1c (%).

### Sensitivity analysis

Stratified analysis could identify which groups benefit from increased ANLR, with results in Table 4 indicating that individuals under 60 years old, White people, non-smokers or previous smokers, those with hypertension or CVD history, and those with an HbA1c of ≥7 are notably benefiting. Interaction results suggested that age and race might interact with ANLR, with no effects from other variables.

**Table 4:** Stratified analyses of the relationships between ANLR and DFU.

Characteristics	ANLR			
	Low 1.47 [0.22,2.05]	High 2.71 [2.05,11.50]	*P* for trend	*P* for interaction
Participants, *n*	765	766		
Age				0.02
≤60	ref	0.28(0.12,0.66)	0.005	
>60	ref	0.81(0.43,1.52)	0.5	
Gender				0.98
Male	ref	0.48(0.23,1.02)	0.05	
Female	ref	0.47(0.22,1.00)	0.05	
Race				0.04
White people	ref	0.34(0.17,0.68)	0.004	
Non-white people	ref	0.81(0.35,1.85)	0.61	
Smoke status				0.65
No/former	ref	0.45(0.25,0.82)	0.01	
Yes	ref	0.44(0.10, 1.99)	0.27	
Alcohol				0.81
No	ref	0.48(0.23,1.00)	0.05	
Yes	ref	0.49(0.21,1.15)	0.1	
Hypertension				0.56
No	ref	0.36(0.12,1.03)	0.06	
Yes	ref	0.50(0.26,0.96)	0.04	
CVD				0.09
No	ref	0.65(0.30,1.41)	0.27	
Yes	ref	0.22(0.09,0.53)	0.001	
HbA1c				0.71
<7	ref	0.49(0.22,1.09)	0.08	
≥7	ref	0.42(0.18,0.97)	0.04	

ANLR, albumin/neutrophil to lymphocyte ratio; DFU, diabetic foot ulcers; CVD, cardiovascular disease.

## Discussion

The DFU is a common and significant complication of diabetes, with approximately 6.3% of diabetic patients worldwide experiencing DFU. The five-year mortality risk for diabetics with DFU was 2.5 times that of those without DFU (23). Around 20% of moderate or more severe DFU infections eventually resulted in amputation (24). These findings suggested that DFU was a major contributor to amputation, considerable decline in life quality, and premature mortality. Growing evidence in recent years indicated a link between DFU, inflammation, and nutritional health. Higher values of NLR, PLR, CRP, and MLR corresponded to greater risks of developing DFU or mortality (21, 25–27). Moreover, typical nutritional indicators, albumin was closely associated with DFU risk (28–30). Meanwhile, inflammation and albumin could also influence each other. Hence, solely focusing on inflammation or nutritional elements was insufficiently comprehensive.

This research initially combined inflammation and nutritional factors, introducing a new DFU predictive marker, ANLR (albumin/NLR). The study first discovered a negative correlation between ANLR and DFU, meaning the higher the ANLR, the lower DFU risk. Every 0.1 increase in ANLR corresponded to a 5% decrease in the risk of DFU. An L-shaped non-linear link was observed based on RCS analysis, with a turning point at 3.09. To the left of the turning point, the trend mirrored the overall pattern, showing a negative correlation between ANLR and DFU. Importantly, on the right side of the turning point, the risk of DFU maintained a stable level, neither increasing nor decreasing with further increases in ANLR. Several factors might contribute to this phenomenon.

Firstly, from the perspective of inflammation. The NLR reflected the inflammatory immune response. High neutrophil levels implied non-specific inflammation, while low lymphocyte levels implied a decline in immune function. Therefore, an increased NLR denoted heightened inflammation (15). It has been demonstrated that patients with diabetes tended to release excessive neutrophil extracellular traps (NETs), causing a substantial increase in neutrophil levels and sustaining inflammation. Neutrophils were readily affected by their programmed cell death (31), impairing wound healing, potentially causing the emergence, advancement, and relapse of DFU. Additionally, research revealed an obstacle in the shift of DFU macrophages from the pro-inflammatory M1 phenotype to the anti-inflammatory, regenerative M2 phenotype, contributing to non-healing wounds. Lastly, chronic wounds constituted a highly oxidative microenvironment. Leukocytes, notably neutrophils, served as rich sources of various reactive oxygen species (ROS) released into the wound environment. This prolongs the healing process of the wound. The evidence pointed out that elevated inflammation levels were associated with higher DFU risks. Considering the data, it was apparent that an elevation in ANLR signified a drop in NLR from an inflammatory perspective, with Table 1 further revealing that NLR was notably lower in the high ANLR group than in the low ANLR group (*p* < 0.0001).

Next, from the aspect of nutrition. Albumin served as a commonly employed factors for evaluating nutritional status in patients. Studies implied that diabetes and its complications involved extensive metabolic disorders (32). Metabolic dysfunctions were intimately connected with diabetic foot, including hypoalbuminemia, dyslipidemia, and obesity, which were risk factors for DFU. Albumin could ameliorate inadequate skin blood flow caused by peripheral artery occlusion, modulate vascular permeability, lessen exudation, and relieve ischemia in diabetic foot. On the other hand, albumin could downregulate the expression of TNF-*α*, IL-1, IL-6, CRP, and MMP-8, reducing inhibitory transport, thus inhibiting inflammatory factors and blocking the “inflammatory storm,” preventing the occurrence of DFU infections (13–15, 33, 34). Albumin played a crucial role in oxidative stress. In ulcer healing, oxidative stress reactions were pronounced, with excessive free radicals causing damage to cells and tissues, thereby delaying the healing process. Albumin, with its potent antioxidant capabilities, could capture free radicals, minimize oxidative damage, and thus supported normal cellular functions and wound healing. Moreover, the amino acid supplied from albumin was crucial for the proliferation of fibroblasts and the synthesis of collagen during the wound healing process. In the early stages of ulcer healing, fibroblasts were crucial as they promote the repair of wounds and the formation of connective tissues. The evidence implied that lower albumin levels correlated with a higher risk of DFU. Summarizing, an elevated ANLR, from the perspective of nutritional factors, suggested increased albumin levels, and Table 1 indicated that the group with higher ANLR has significantly greater albumin levels than the group with lower ANLR (*p* < 0.0001).

Why, then, did an increase in ANLR to the right of the inflection point not further diminished the risk of DFU? Drawing from past research, we hypothesized that: (1) Repair of wounds or traumas necessitated adequate inflammatory involvement. Excessively high or low inflammation levels might impair wound healing. (2) Further escalation of ANLR signified a substantial increase in albumin and/or a significant drop in NLR. Together, these alterations promoted a decline in inflammation levels, going beyond what was considered appropriate. (3) Integrating the two preceding points, the risk of DFU could not further decline with the continuous increase in ANLR. Overall, from a broader perspective, the risk of DFU decreased with the increase in ANLR, but upon finer analysis, this negative correlation has an inflection point effect. Beyond a ANLR of 3.09, further increased no longer reduce DFU risk.

This study presented three key benefits: first, a large number of samples and reliable, rigorous data sources. Second, the study accounted for the influence of various confounding factors, employing multi-factor logit regression and sensitivity analysis to reduce their impact as much as possible. Third, it constructed a new predictive indicator that comprehensively evaluated the effects of inflammation and nutritional status on DFU risk. Furthermore, the elements (albumin and NLR) were broadly utilized in clinical settings, easily accessible, and beneficial for clinical applications.

It must be acknowledged that this study stilled has some limitations. Specifically, as an observational study, the inherent characteristics of the study did not allow for a clear establishment of causality between ANLR and DFU risk, similar to what was possible in prospective clinical studies. Future large prospective studies were required to substantiate the findings of this research. Moreover, although multiple methods were used to control confounding factors, unknown biases persisted.

## Conclusion

To put it succinctly, the research not only presented a novel comprehensive index for predicting DFU but also clarified the impact of ANLR on DFU risk. Generally, there existed a negative association between them. As ANLR raised, the risk of DFU diminished. However, a more detailed analysis revealed that this negative correlation has an inflection point effect. Once ANLR surpassed the inflection point, further increased in ANLR did not continue to decrease DFU risk. Furthermore, each 0.1-unit increase in ANLR resulted in a 5% reduction in DFU risk, enabling clinicians to more accurately assess individual risks of DFU and aiding in earlier detection and intervention. Therefore, for diabetic patients with low serum albumin, appropriate supplementation of albumin was crucial. Additionally, maintaining the NLR at an appropriate level should not be overlooked. Ultimately, the constituent elements of ANLR (albumin and neutrophils, lymphocytes) were extensively utilized and readily available in clinical practice, promising significant prospects for future clinical applications.

## Data Availability

The original contributions presented in the study are included in the article/Supplementary material, further inquiries can be directed to the corresponding author.

## Footnotes

^1^ www.cdc.gov/nchs/nhanes/

^2^ http://grants.nih.gov/grants/policy/hs/hs_policies.htm
