# Supplementary material for: Association between nutritional inflammation index and diabetic foot ulcers: a population-based study
Source: Front Nutr. 2025 Jan 24;12:1532131. doi: 10.3389/fnut.2025.1532131 (PMC11802432; doi:10.3389/fnut.2025.1532131)
Supplement: Supplementary file 1 [file Data_Sheet_1.doc]

**Supplementary Tables 1. Threshold effect analysis of ANLR on DFU**

**Supplementary Tables 2. Threshold effect analysis of ANLR on DFU**

**Supplementary Tables 3. Relationships between ANLR and DFU**

**Supplementary Figure 1. Non-linear relationship between ANLR and DFU. Adjusted for age, gender, race, smoke and alcohol status, hypertension, CVD, HbA1c, diabetes duration, and anti-diabetic treatments (hypoglycemic agents and insulin).**

**Supplementary Figure 2. Comparison of two ROC curves constructed based on ANLR and three other risk factors (age, diabetes duration, and HbA1c levels).**

**Supplementary Tables 1. Threshold effect analysis of ANLR on DFU**

|  | **Incidence** | |
| --- | --- | --- |
|  | **Per 0.1U increment** | ***P*** |
| <2.05 | 0.89(0.82,0.96) | 0.01 |
| >2.05 | 1.00(0.95,1.04) | 0.85 |

Abbreviations: ANLR, albumin/neutrophil to lymphocyte ratio; DFU, diabetic foot ulcers; CVD, cardiovascular disease.

Model was adjusted for ANLR, age, gender, race, smoke status, alcohol, hypertension, CVD, and HbA1c (%).

**Supplementary Tables 2. Threshold effect analysis of ANLR on DFU**

|  | **Incidence** | |
| --- | --- | --- |
|  | **Per 0.1U increment** | ***P*** |
| <4.00 | 0.93(0.89,0.97) | 0.002 |
| >4.00 | 0.82(0.56, 1.21) | 0.29 |

Abbreviations: ANLR, albumin/neutrophil to lymphocyte ratio; DFU, diabetic foot ulcers; CVD, cardiovascular disease.

Model was adjusted for ANLR, age, gender, race, smoke status, alcohol, hypertension, CVD, and HbA1c (%).

**Supplementary Tables 3. Relationships between ANLR and DFU**

| **ANLR** | **OR, 95%CI** | | |
| --- | --- | --- | --- |
|  | **Crude** | **Model 1** | **Model 2** |
| Low | ref | ref | ref |
| High | 0.45(0.26,0.79) | 0.44(0.24,0.81) | 0.44(0.24,0.83) |
| Per 0.1 U increment | 0.96(0.92,1.00) | 0.96(0.92,1.00) | 0.96(0.92,1.00) |
| ***P* for trend** | 0.01 | 0.01 | 0.01 |

Abbreviations: ANLR, albumin/neutrophil to lymphocyte ratio; DFU, diabetic foot ulcers; CVD, cardiovascular disease.

Model 1: adjusted for age, gender, and race. Model 2: model 1+ adjusted for smoke status, alcohol, hypertension, CVD, HbA1c (%), diabetes duration, and anti-diabetic treatments (hypoglycemic agents and insulin).


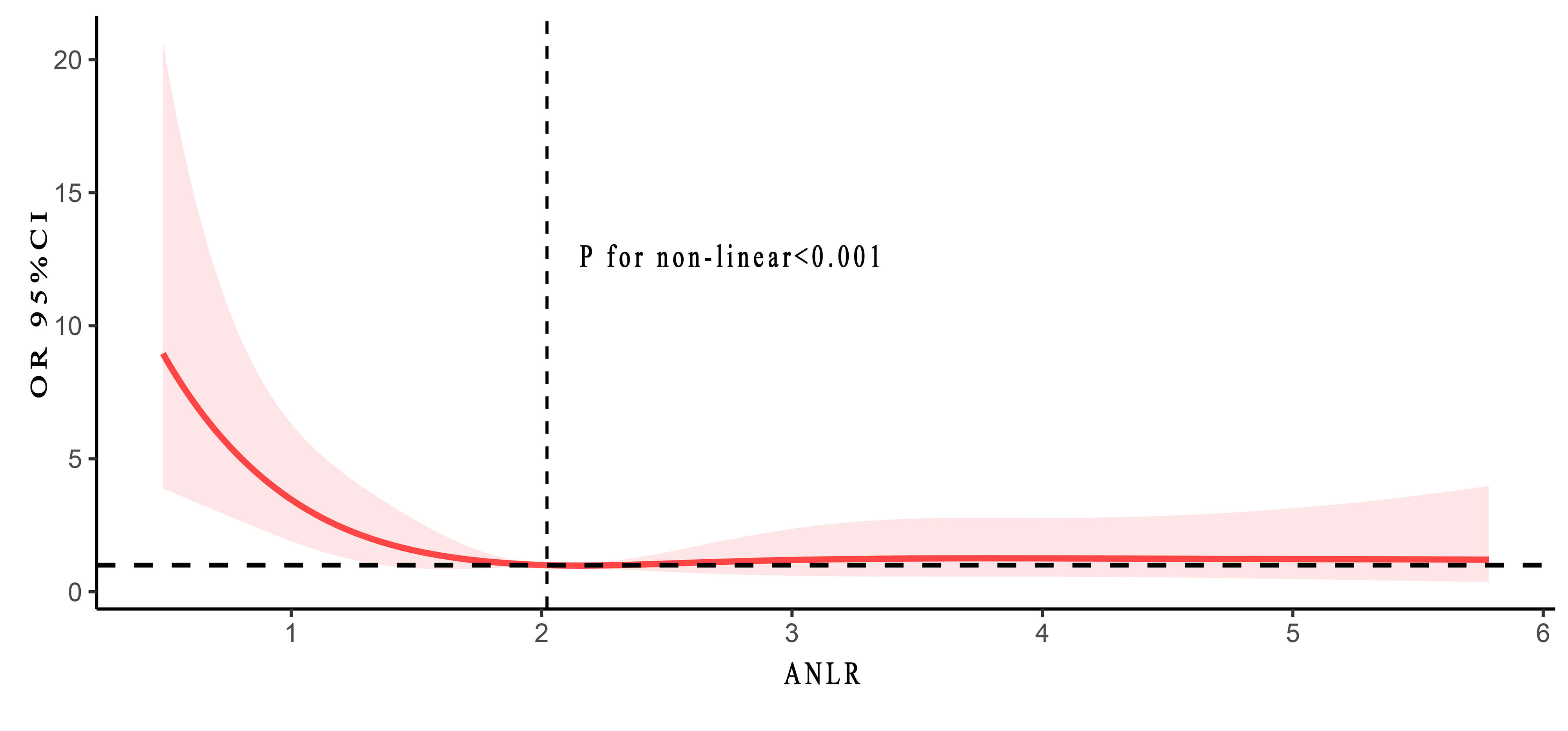


**Supplementary Figure 1. Non-linear relationship between ANLR and DFU. Adjusted for age, gender, race, smoke and alcohol status, hypertension, CVD, HbA1c, diabetes duration, and anti-diabetic treatments (hypoglycemic agents and insulin).**


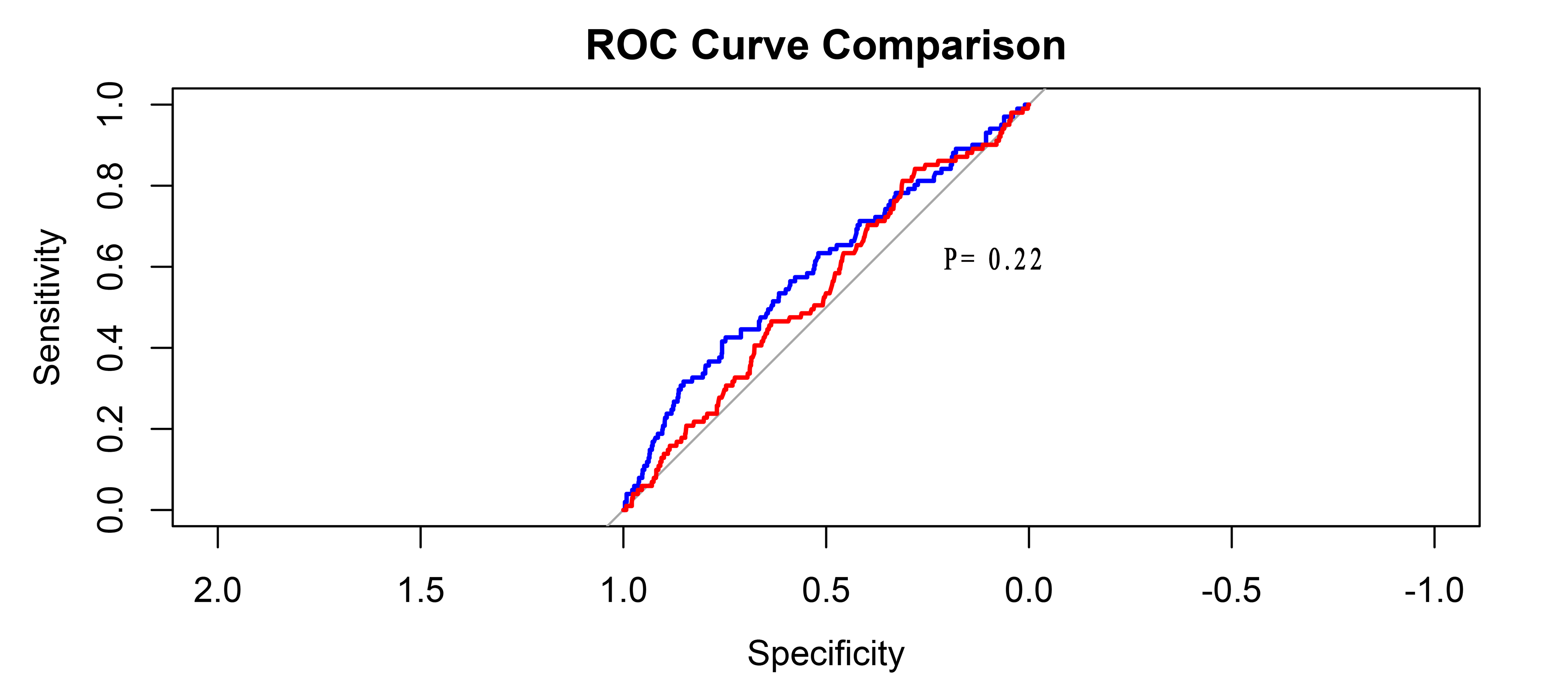


**Supplementary Figure 2. Comparison of two ROC curves constructed based on ANLR and three other risk factors (age, diabetes duration, and HbA1c levels).**
